# 

**Published:** 1890-10-04

**Authors:** 


### The Hospital

A Weekly Institutional Journal of

### Science, Medicine, Nursing, and Philanthropy.

EDITED BY

HENRY C. BURDETT,

AUTHOR OF "HOSPITALS AND THE STATE"; "PAY HOSPITALS OF THE WORLD"; "COTTAGE HOSPITALS, GENERAL, FEVER, AND CONVALESCENT WITH FIFTY BEDS AND UNDER: THEIR ORGANISATION, MANAGEMENT, AND WORK"; "BURDETT'S HOSPITAL ANNUAL"; "HELP IN SICKNESS"; "HELPS TO HEALTH"; ETC., ETC.

ACTING EDITOR

GEORGE W. POTTER, M.D.,

AUTHOR OF "MINISTERING WOMEN" PAPERS ON "BACTERIOLOGY"; "SCARLET-FEVER"; "RHEUMATISM"; "HYPNOTISM"; ETC. ETC

INDEX TO VOL. IX.

(4th October, 1890, to 28th March, 1891.)

FOR 1891.

LSCO

5/a 68

LONDON:

PUBLISHED BY "THE HOSPITAL" (Limited), 140, STRAND, W.C.

W1
H069X

LONDON:

W. H. AND L. COLLINGRIDGE, "CITY PRESS,"

148 AND 149, ALDERSGATE STREET, E.C.

### INDEX TO VOL. IX. 1890-91.

Aberdeen Children's Ailing Home, 252
Accounts, Uniformity in Hospital, 260
Acland, Sir Henry, on Medical Women's Rights, 121
Addenbrooke's Hospital, Quarterly Court, 288
Ærated Waters for Hospitals, 269
Aqua de Dios, Leper Community of, 188
Alcohol and Childhood, 41
Alexandra Hospital's Bournemouth Branch to be Enlarged, 312
Ambulance Instruction in Manchester and the Police, 324
——— Service, London's Street, Opening of Station at Royal Exchange, 325
America, Physical Education in, 67
American and English Girls, 190
Aneurysm, Intra-cranial, 176
Animals, Royal Society for Prevention of Cruelty to, the Society's Work, 325
Anthrax, Death from, 205
Antipyrin, Poisoning by, 317
Antisepsis in Dyspepsia, Intestinal, 351
Antiseptic Spray, The, a Bone of Contention, 1
——— Surgery, 328
——— Treatment of Wounds, A New, 146
Anti-Vaccination at Tunbridge Wells, 290
Aphonia, Hysterical, 237
Appeals, The Season of, 198
——— Charitable, Mr. Andrew Tuer on, 284
Appendicitis, 352
Aprosexia in Children, 339
Areas from which London Hospital Patients are Drawn, 320
Army and Navy Pensioners' Employment Society, 24, 306
Assessment of Hospitals and the London County Council, 344
Ataxies, Anomalous, 293
Attacks on Hospitals, Unwarranted, 272
Australian Hospitals, 124

Beer at Christmas, Guardians on, 204
Begging, Profitableness of, 173
Belfast Royal Hospital, 140
Bequests to Charities, 372
Bible Women and District Nurses' Mission, 336
Bideport Infirmary, an Endowment for, 190
Birkenhead Hospital Scandal, 109
Birmingham Committee on Hospital Reform, 57, 93, 125; Report, 348
——— General Hospital to be Rebuilt, 42, 300
——— Hospital Abuse at, 40
——— Reform Inquiry, 188
Birthrates and Population, 34
Blackpool Hospital, 8.
Bladder, Diseases of, by Mr. Reginald Harrison, 75
Bolingbroke House Pay Hospital, Proposed New Wing for, 250
Bookkeeping, Medical, 293
Booth, "General"—the Effect of his Fund on the Hospitals, 244
Boscombe Cottage Hospital, Bazaar at, 172
Boston Hospital, Mr. Wheeler's Work at, 174
Bournemouth, Royal Victoria Hospital, Annual Meeting, 277
Bradford, Children's Hospital, 40
Brandreth, Mr., on Mendicancy as a Trade, 173
Breakfast, About, 278
Bristol General Hospital, 156
——— Annual Meeting, 360
——— Extension of, 348
——— Medical School, 97
——— the Hospitals of, 98
Bristowe, Dr., on Heart Disease, 59
——— on Nervous Diseases, 96
——— Mr. L. S., on the Legal Restrictions on Gifts to Charities, 289, 296
British Artists, A Hint for, 15
Bromethyl, 268
Brompton Consumption Hospital, 156
——— before the Lords' Committee, 366, 379
Bromsgrove Cottage Hospital, 93
Bruce, Dr. Mitchell, on the Treatment of Cough, 291
Bull Dog, No Gentleman Keeps a, 15, 46
Burdett, Mr. Henry C., on Nurses' Food, Work, and Recreation, 135, 141
Burdett-Coutts, Mr., on Hospitals, 361
Burial Reform and the Church of England, 205
Burnett, Mr. Egerton, Offers to Build a Hospital for Wellington, 235
Burns, Death from, 27

Cambridge, Body and Mind at, 331, 371, 374
——— University Medical Society, 176
Cancer, by Dr. W. Russell, 159
——— Cure of, and Count Mattei, 271
——— Hospital, Brompton, 336
——— The Etiology of, 280
——— The Tomato and, 168
Canterbury Dispensary, 300
Cataract, 209
Chaplains of Hospitals, 126
Charing Cross Hospital, Annual Report of, 324
——— Before the Lords' Committee, 330
Charity and Charity Organization, 362
——— Organization Society as Collector for Charities, 214
——— Plea Against its Undue Growth, 266
Charities, The Legal Restrictions on Gifts to, 289
Cheadle, Dr., on Rheumatic Affections, 175
Cherry Blossom Soap, 147
Chest, Royal Hospital for Diseases of the, 361
Child Labour, A Crisis in, 374
Children, East London Hospital for, 336
Children's Convalescent Homes, 189
Choirs in Hospitals, 66
Cholera, Cure for, 338
——— Japanese Officials and, 264
——— The Approach of, 253
Chorea, 279
Christchurch Workhouse Scandal, 57
Christmas, The Poor Man's, 181
City of London Truss Society, 300
Clap-trap Criticism, 32
Clark, Sir Andrew, on the Convulsive Cough of Puberty, 192
——— Work and Food, 154
——— Sketched by the "Gentlewoman," 252
Clergy, Corporation of the Friends of the, 306
Climatology, Mr. B. Thornton on, 60
Club Foot, Treatment of, 240
Cocoa and Chocolate, 114, 146
Coffee, Cocoa, or Tea, 146
Coldstream Cottage Hospital, 250
Collectors' Commission, Fifteen per Cent., 86
Consumers' League, The, 169
Consumption, A French Cure for, 266
Convalescent Home for Suffolk, 189
Cook, Captain, by Walter Besant, 138
Corsets or not, 161
Cough of Puberty, The Convulsive, 192
——— On the Treatment of, 291
Criminal Anthropology, Some Notes on, 309
——— The, 247
Croydon Board of Guardians and Diphtheria, 141
——— Diphtheria at, 142
Crude Science, 149

Deaf and Dumb Females, British Asylum for, 265
Death Certificates and Murder, 14
December, 162
Deglutitio Impedita, 12
Dental Chemistry and Metallurgy, by C. Mitchell, M.D., 317
——— Hospital of London, 360
Derby and Derbyshire Infirmary, 92
——— Lord, on Hospitals, 228
Derbyshire General Infirmary to be Re-constructed, 251
Derby Union Infirmary, West, Rebuilding of, 372
Devonport Royal Albert Hospital, 140
Devonshire Hospital and Buxton Bath Charity, 264
Diabetic Coma, 292
Diphtheria at Cheam, 74
——— Public Schools, 101, 137, 206
——— Intubation in, 177
——— Loeffler on, 237
——— Treatment of, 267
Dispensaries in Birmingham, Provident, 188
Doctors, American, 360
——— and the Peerage, 227
Doctor's Armchair, The :—
Life Problem at Forty, The, 69
Can Science Resolve the Doubt ? 87
Medical Women's Rights, A New Champion of, 121
Has a New Day Dawned ? 151
Koch, Jenner, and Pasteur, 167
Charity's Much-injured Name, 183
Kosher and Trifa, 199
Poisons as Required, 229
Winter Feeding, 245
Lupus and Tuberculosis, 261
Science in Straits, 273
A Dream : Perhaps a Delusion, 285
Some Risks of Middle Age, 333
A Feat to be Considered, 357
A Counsel of Despair, 297
The Registration of Midwives, 321
The Gnat and the Camel, 345
A New Interpreter of Faith, 369
Doctor's Difficulties in Town and Country, The, 319
Doctor's Pulpit, The :—
Props and Buttresses, 3
Are Lady Doctors Clever ? 19
How to Keep Well, 35
The Perfection of Human Happiness, 51
Doncaster Infirmary, 26
Dublin Children's Hospital, 140
Dudley Guest Hospital, 157
Dufferin's Fund, Lady, Annual Report of, 204
Dundee, New Hospital for, 234
Durham County Hospital, 372

Ear, Diseases of the, 257
Ears, Noises in the, 210
East End Mothers' Home, 265
Edinburgh Royal Infirmary, Annual Meeting, 251
Education, Physical, in America, 67
Eggs, Act for the Protection of Wild Birds', 373
Electro Therapeutics, 293
Empyema, The Etiology of, 176
Encephalocele, the Surgical Treatment of, 267
Encephalitis, Primary Acute, 292
English Medical Books, The Two Oldest, 88
Enteric Fever, Starvation in, 27
Epilepsy, Reflex, from Dental Caries, 292
Epileptics, Homes for, 324
Erysipelas, New Methods of Treatment, 254
Erythro-Melalgia, 375
Escholtzia Californica, 329
Essex and Colchester Hospital, Annual Report of, 324
Euphorine, 293
Eyes and Noses, 89

Fawcett, Mrs., on Socialism, 105
Femur, Growth of, after Resection of Knee Joint, 239

Fernie, W. T., M.D., on the Tomato and Cancer, 168
Field Lane Institution, An Account of, 313
Flatfoot, 112, 241
Fogs and Death Rate, 188
Foreign Body, Spontaneous Expulsion of a, from the Bronchi, 267
Foundling Hospital, Christmas at, 234
Fowler, Dr. J. Kingston, on Diseases of the Lungs and Pleura, 191
French Hospital, Bazaar in aid of, 156
Friendly Societies and Project for Suffolk Convalescent Home, 189

Ganglions, 240
Gangrene and Tetanus, Association of, 280
Garden, a Hospital, 185
Gastric Ulcer, 327
German Hospital, Annual Court of, 300
——— Universities and English Students, 50
Giving, the Luxury of, 217
Glasgow Dental Hospital, 372
——— Maternity Hospital, Annual Report of, 172
——— Royal Infirmary, Annual Report of, 300
——— ——— to take over the Ophthalmic Institution, 300
——— Victoria Infirmary, Progress of Building, 172
Gonorrhœal Arthritis, Gout and Rheumatoid Arthritis, 175
Gorleston Cottage Hospital, Need of Funds, 211
Gosse, Philip Henry, 252
Great Northern Central Hospital, 336
Guy's Hospital, Improvements in the Medical School, 312

Hampstead Home Hospital, Annual Report of, 373
Harrison, Mr. Reginald, on Diseases of the Bladder, &c., 75
Heart Disease, by Dr. Bristowe, 59
——— Some Functional Disorders of the, 315
Hemiatrophia facialis, 111
Hepatic cirrhosis, 27, 207
Herbal Simples, the History and Capabilities of :—
Club Moss, 6
Clover, 37
Comfrey, 37
Coltsfoot, 70
Cowslip, 122
Cress, 186, 200
Currants, 232
Daisy, The Common, 246
Dandelion, 346
Dill, 370
Hertford British Hospital in Paris, 106
Herpes, 376
Holidays by the Sea, 4
Homes for Little Boys, Account of the, 349
Hospital Administration, Advance in, 19
——— Chaplains, 126
——— Choirs, 66
——— Committees, Mixed, 58, 61
——— for Sick Children, Festival Dinner of, 373
——— Nurses, 123
Hospital Saturday at Bolton, 8
——— Hastings, 8
——— Worcester, 109
——— Manchester and Salford, 156
——— Melbourne, 188
——— Norwich, 204, 234
——— Fund, Metropolitan, Annual Meeting, 360
——— Metropolitan, Disagreement, 308
Hospital Sunday at Newcastle, 8
——— Liverpool, 108
——— Manchester and Salford, 156
——— Melbourne, 188
——— Norwich, 204
——— Hull, 235
——— Fund, Metropolitan, 158, 205, 275
——— and Uniformity of Accounts, 260
——— Special Preachers suggested for, 166
Hospital Work During 1890, 215, 231
Hospitals Association, 141
——— Meeting of, at Middlesex Hospital, 173
——— for all, 338
——— from the Statistical point of View 182
House on Wheels, A, 302
——— Surgeons of Hospitals, and Treatment of Doubtful Cases, 266
——— Ventilation and Warming, 20
Hull Orthopædic Hospital, 8
——— Royal Infirmary, the Working Men's Committee, 276
——— Victoria Hospital for Children, 72

Humphry, Professor, on the Human Form, 212
Hutchinson, Mr. Jonathan, on Senile Diseases, 43
——— on Lupus, 250
Hypnotism, 202

Ignatia Amara, 341
Ill Health and Crippled Powers, 206
Infants' Foods, 241, 281
Infirmaries, Students in, 58
Infirmary Medical Superintendents' Society, The, 178, 230
Inoculation for Crime, 326
Insane, Dr. Greene on Hospitals for the, 337
———Metropolitan Hospital for the, 366
Insane, The Story of the, from Year to Year :—
Berks County Asylum, 9
Nottingham County Asylum, 9
Earlswood Asylum for Idiots, 9
Northumberland County Asylum, 29
North Riding Asylum, York, 41
Salop and Montgomery Asylum, 41
Newcastle-upon-Tyne Asylum, 41
Warwick County Asylum, 57
Devon County Asylum, 57
Lancashire County Asylum, Prestwich, 57
Argyll and Bute District Asylum, 73
Perth District Asylum, 73
Kent County Asylum, Canterbury, 73
Ipswich Borough Asylum, 73
Wilts County Asylum, Devizes, 93
Lancaster County Asylum, Lancaster Moor, 109
East Riding Asylum, Beverley, 248
Perth Royal Asylum, 248
Suffolk County Asylum, 248
Intra-cranial Vessels in Children, Diseases of the, 375
Intratracheal Injections, 237
Invalid Dress, A New, 13
Iodides and Salicylates, 111
Iodoform, Treatment of Cold Abscess by, 210

Japan, Cholera in, 264
Johns Hopkins University, Medical Women at the, 283
Jubilee Institute for Nurses, Report of the, 368

Kidd, Dr. Percy, on Tubercular Affection of the Larynx, 127
King's College Hospital, 336
——— before the Lords' Committee, 378
——— Entertainment at, 276
Kirkcaldy, New Hospital opened at, 140
Knee Joint, Chronic Disease of the, in Children, 317
Koch and the Consumption Cure, 216
——— and Virchow, 259
——— Dr., 305
——— on Tuberculosis, 129
Koch's Cases and other People's, 110
——— Cure and the Dublin Hospitals, 251
———Dr. Theodore Williams on, 107
——— Discovery and British Medical Men, 125
——— what to Expect from it, 85, 117, 158
——— Notes of Warning, 139
——— Remedy, On the Therapeutical Action of, 363

Lady Doctors, Dr. Sarah Post on, 174
Lancaster Infirmary Committee, 73
——— Workmen and the, 204
Lanolin and Vaseline, 268
Larynx, Tubercular Affection of, by Dr. Percy Kidd, 127
Leeds Infirmary, Changes in Management of the, 172
——— New Wing Building at, 360
Legal Restrictions on Gifts to Charity, 296
——— Bill to Remove the, 372
Leicester Infirmary, 372
——— Financial Condition of, 276
Leper Village in Colombia, 188
Leprosy, a Case of Supposed Indigenous, 95
——— Commission, The, 360
Library, the Greatest Medical, in the World, 78
Life Insurance and Murder, 14
——— Preservation from a Business Point of View, 54, 75, 96, 112, 128, 145, 160
——— Problem at Forty, The, 69
Light Accident at St. Thomas's Hospital, 348
Lincoln County Hospital, Annual Meeting, 288
Linton, Mrs. Lynn, on the Vice of Pity, 218
Liver, A Wandering, 210
——— Hydrated Cysts of, 211
——— Resection of the, 28
Liverpool Royal Infirmary, 92
London Hospital, a Word to the Governors of the, 142

London Hospital, The, and the Attacks on its Matron, 142, 143
London's Lunatics and Provision for Them, 74
London Poor, The, 275
——— Post Graduate Course at Brompton Consumption Hospital, 127, 143
——— Post Graduate Course at Paddington Infirmary, 43, 59, 75, 96, 112, 128, 145, 160
Lords' Select Committee and the Hospitals, 154, 243, 278, 306, 342, 366
——— Suggestions to the, 243
——— Middlesex Hospital Examined, 318
——— St. Mary's Hospital Examined, 330
——— Charing Cross Hospital Examined, 330
——— Improvements in the Methods of the, 333
——— Royal Free Hospital Examined, 354
——— Metropolitan Hospital Examined, 354
——— Brompton Hospital for Consumption Examined, 378
——— King's College Hospital Examined, 378
——— and Domestic Scandal, 16
Lückes, Miss Eva C. E., 21
Lucknow, New Hospital for Women at, 324
Lunatic Asylums, Voluntary Patients in, 94
Lungs and Pleura, The Physical Diagnosis of diseases of the, 191
Lupus, Mr. Hutchinson on, 250
——— The Relation of, to Tubercle, 176
Lyddon, Dr., The Murder of, 205

Mackinnon Memorial Hospital Committee, 235
Madeira Seamen's Hospital, 173
Madness, What is it? 314
Malignant Diseases, Treatment of, 304
Manchester Consumption Hospital, Proposed Enlargement of, 361
——— Infirmary to be Enlarged, 250
——— Offer by the Whitworth Trustees to, 336
Marlborough College, Diphtheria at, 101, 137
Marshall, Mr. John, F.R.S., Death of, 236
Matron, A Celebrated, 21
Mattei, Count, and the Cancer Cure, 271
Medical Books, The Two Oldest English, 88
——— Library, The Greatest in the World, 78
——— Profession, Qualifying Age for Entry into, 134
——— Racks and Thumbscrews, 110
——— Schools, Entries Winter Session 1890-1891, 77
——— in the United States, 103
——— Syndicates, 123
——— Women in the Johns Hopkins University, 343
——— in England, 343
Membrana Tympani, Spontaneous Restoration of the, 210
Mental Derangement and Outward Surroundings, 15
Menthol in Diphtheria, 111
Metropolitan Asylum Board, 156
——— Statistics, 124
——— Free Hospital Before the Lords Committee, 354
——— Hospital, Annual Meeting, 288
Microscope in Medicine, The,—
I. Introductory, 231
II. The Use of the Microscope, 262
III. The Microtome and Hardening Specimens, 274
IV. Cutting Sections, 286
V. Staining Sections, 298
VI. Mounting Specimens, 311
VII. Methods of Staining Sections of the Nervous System, 323
VIII. The Examination of Sputum, 334
IX. The Examination of Sputum, 347
X. The Examination of Urinary Deposits, 358
Middlesex Hospital, 336
——— before the Lords' Committee, 318
Midwives' Registration Bill, 215, 295, 307, 314, 354, 378
Milk and Typhoid in Edinburgh, 360
——— Mothers', 241
Mimpriss Bequest, 8
Modern Pathology, The Relation of, to Practical Medicine, 104
Montreal, Notre Dame Hospital, 124
Moore, Sir W., on Tropical Colonization, 316
Morley House Convalescent Home, Proposed Extension of, 172
Monat, Dr., on Hospitals, 182

Nasal Obstruction and Mouth Breathing, 293
National Pension Fund, 42, 118, 134, 150, 215

Nurses, Male, 35, 50
——— the Registration of, 215, 342

October, 29
Oldham Infirmary, Annual Report of, 312
One-roomed Life in London, 290
Ophthalmia, Granular, 329
——— Membranous, 352
Orphan Working School, An Account of the, 313
Ouabaine, 256
Out-patient Question, The, 10

Paignton, New Cottage Hospital for, 264
"Pall Mall Gazette" attacks the London Hospital, 165
Pancreas, Disease of the, 11, 339
Paris, The Hertford British Hospital in, 106
Parkin Bequest, 360
Pasteur and Hydrophobia during 1890, 216
——— and Rabies, 201
Patents, Some Curious, 55
Patients' Dinners, 26
Pathological Society of London, 159
Pathology, The Relation of, to Practical Medicine, 104
Paupers' Food at Worcester, 158
Pennyroyal, 268
Pension Fund, The, and the Hospitals, 42, 118, 134, 150
Pensions for Hospital Workers, 118
Peritonitis, Tubercular, Surgical Treatment of, 177
Perityphlitis, 352
Philanthropists, The, Who are They? 213
Philanthropists' Vade Mecum, The, 219
Physical Education, 278
Physicians, The College of, and the Status of Army Doctors, 355
Pittsburgh, U.S.A., Department of Public Safety, 54
Pity, The Vice of, 218
Pneumonia, Cold Treatment of, 255
Poisoning by Colchicum, 75
Poole, Cornelia, Hospital at, 141
Poor, Association of Friendly Workers amongst the, 362
——— Men's Houses, 42
Portobello Fever Hospital, 10, 235
Post, Dr. Sarah, on THE HOSPITAL's Views as to Lady Doctors, 174
—— Graduate Course:—
Gonorrheal Arthritis, Gout and Rheumatoid Arthrit s (Dr. Cheadle), 75
Physical Diagnosis of Dise ses of the Lungs and Pleura (Dr. J. Kingston Fowler), 191
—— Graduate Course, The, 291
Press, The, and the Enquiries into Hospitals, 35 6
Princess Louise Home, Annual Report, 264
Propo-e, May a Woman, 87
Prostate Gland, Hypertrophy of the, 238
Pulmonary Phthisis, by Dr. Percy Kidd, 143

Quain, Dr., 236

Rabies, 201
Recreation in Relation to the Health of the People:—
I.—Introductory, 270
II.—Beginning at the Bottom, 282
III.—Working Up, 299
Rectal and Anal Surgery, 3[illegible]3
Reform of Hospitals, Birmingham Committee on, 188
——— Committee's Report on, 348
Refuges for Homeless and Destitute Children, National, 265
Registration of Nurses, 215
Reigate and Redhill Cottage Hospital, 109
Religion Oblige, 110
Rentoul, Dr., and Midwives' Registration, 174
Retina, Treatment of Detached, by Iodine, 75
Reviews:—
The Drink Question (Dr. Kate Mitchell), 38
A Short Manual of Personal and Domestic Hygiene (A. T. Schofield), 38
Health and Comfort in House Building (Drysdale and Hayward), 20
The Modern Malady (Cyril Bennett), 53
The Demoniac (Walter Besant), 119

Reviews:—
Captain Cook (Walter Besant), 138
Pasteur and Rabies (T. M. Dolan, M.D.), 201
The Criminal (Havelock Ellis), 247
Gruber on Ear Diseases, 257
Nasal Obstruction and Mouth Breathing (Dr. Spicer), 293
Practical Electricity in Medicine and Surgery (Liebig and Rohé), 293
Medical Bookkeeping, 293
Dental Chemistry and Metallurgy (C. Mitchell, M.D.), 317
Rectal and Anal Surgery (E. Andrews, M.D., and E. W. Andrews, M.D.), 353
Rheumatism, by Dr. Cheadle, 144
Rickets, Treatment by Phosphorus, 279
Rickety Limbs, Removal of Deformity of, 210
Robertson-Stewart Hospital, Rothsay, Annual Meeting, 276
Rosebery, the late Lady, 142
——— and the Hospitals' Association, 141
Royal Albert Orphan Asylum, Its Work, 349
———Berks Hospital, Special Court of Governors, 276
———Free Hospital and Festival Dinner, 373
——— Annual Meeting of the, 312
——— Ball in Aid of, 173
——— before the Lords' Committee, 354
——— Changes in Staff, 276
——— Medical Women and the, 371, 374
———Hospital for Children, Annual Court, 348

St. Bartholomew's Hospital, Sanitary Condition of, 318, 361
St. George's Hospital before the Lords' Committee, 306
St. John of Jerusalem, Hospital of, Annual Report, 348
St. Mary's Hospital, Paddington, Annual Meeting of, 372
——— before the Lords' Committee, 330
———Roman Catholic Hospital, Rochester, New York, Fire at, 324
St. Monica's Home Hospital, Brondesbury, 251
St. Thomas's Hospital before the Lords' Committee, 306
Saline Sub-cutaneous Injections, 27
Salol, Action of, on the Kidneys, 177
Salt as an Antiseptic, 177
Sanitation at Huddersfield, 350
Saundby, Dr., on Hepatic Cirrhosis, 207
Scarlatina without Fever, 237
Schools and Diphtheria, 101
———, Manual Training in, 5, 23
Sciatica, 303
Science, Pugilistic, 1
Seaford Seaside Convalescent Hospital, 140
Seamen's Hospital before the Lords' Committee, 362
——— Society, Annual Court of, 324, 326
Seigel's Curative Syrup and a Leeds Doctor, 205
Senile Diseases, Mr. Jonathan Hutchinson on, 43
Shanklin, New Surgical Home for Children at, 312
Sheffield General Infirmary and Dr. Koch, 234
——— Infirmary, 108
——— Public Hospital, Gifts and Bequests to, 92
Shipley, Sir Titus Salt's Hospital at, New Departure at, 312
Singer, Mr. A. M., Gives a Cottage Hospital to Paignton, 264
Sleeping Sickness in a London Hospital, 172
Smoking, Juvenile, the Suppression of, 362
Snake Bite, Cure for, 338
Socialism, Mrs. Fawcett on, 105
Soda, Dithiosalicylate of, 377
Spicer Dr. Scanes, on Nasal Obstruction, 293
Spina Bifida, 239
——— Ventosa, 239
Starvation and Indigestion, 94
Starving into Health, 28, 94
Statistical Society and Hospitals, 182
Strychnine as an Antidote, 281
———Poisoning, 268
Student, What says the, 65
Students Chronic, 374
———in Pauper Hospitals, 58
Sunderland New Infectious Hospital, 8

Sunlight and Disease, 304

Surgery, Antiseptic, 340

Sussex County Hospital, 193

Tabies, A New cause of, 292
Tattlers, 102
Tea, Coffee, or Cocoa, 146
Thirsk, Cottage Hospital, 8
Thomson, Archbishop, and Working Men, 218
Thought Production, Explanation of, required, 206
Thyroid Grafting, 28
Tongue, Fissures of the, 340
Tonsilitis, The Pathology and Surgical Treatment of Suppurative, 377
Torquay, Okendon Convalescent Home, 156
Torticollis, 364
Toxteth, New Workhouse for, 251
Tropical Colinization, some Medical Aspects of, 316
Tubercle, Facts about, 94
Tubercular Disease of the Bones and Joints, 255
Typhlitis, 352
Typhoid and Milk in Edinburgh, 360

University College Hospital before the Lord's Committee, 342
Urticaria, 207

Veratrum viride, 305

Vertigo, Cardiovascular, 292

Virchow and Koch, 259

Walmsley, F. H., M.D., on a Metropolitan Hospital for the Insane, 366
Weil's Disease, 111
Wellington, New Cottage Hospital for, 235
West End Hospital, 348
Westminster Hospital before the Lords' Committee, 342
Wethered, F. J., M.D., on the Microscope in Medicine, 231, 262, 274, 286, 298, 311, 323, 334, 347, 358
Whitford, Mr., Appointed Secretary of Shadwell Children's Hospital, 204
Wigan Infirmary, New Wing, 8
Williams, Dr. Theodore, on Koch's Cure, 197
Wilson, Miss, on Nursing in Workhouses, 173
Winter Session at the Medical Schools, 26
Witney, New Convalescent Home at, 189
Wives, The Duty of, 10
Women and Children, Slaughter of, 236
——— and Politics, A Hampshire Governess on, 326
——— at the Johns Hopkins' University, Medical, 282
——— Medical, and the Royal Free Hospital, 371
——— on Hospital Committees, 58, 61
——— The Medical Education of, 39
Women's Hospital, Medical Institute at, 188
——— Rights, A New Champion of Medical, 12[illegible]
Worcester Convalescent Fund, Women's Branch of the, 277
——— Guardians and Paupers' Food, 158
——— Infirmary, Financial Condition of, 348
——— Ophthalmic Hospital, Annual Meeting of, 277
——— Victoria Kitchens, 156
Words of Consolation:—
Loneliness, 2
Thanksgiving, 18
Work and Food, by Sir Andrew Clark, 154
Workhouse Infirmary Nursing Association, The, 153
Working Men's Committees and the Hull Royal Infirmary, 276
——— Societies and the City Truss Society, 300
——— Women in Large Towns, 30, 36, 68, 90, 119, 152, 184, 294
Workmen and the late Archbishop of York, 218
——— Surbiton Cottage Hospital, 36[illegible]

York County Hospital, Satisfactory Financial Condition of, 372

Zenana Medical College, 14[illegible]

### THE SPECIAL NURSING SUPPLEMENT (MIRROR).

Accusations against Hospitals, Rash, cxliii
American Notes, l, cxvi, cxxviii
Appointments, cxv, cxxiv
Atcham Guardians Appoint Imbeciles as Nurses, lxxxix
Ashton-under-Lyne, Borough Hospital at, lxxxiii
Asylum Attendants, xxiii, xxxvii, xxxix, lxii, lxxxvi
Australia, Notes from, vii, xxv, xliv, lxxxv, cx, cxxii, cxlvii
Aylesbury, An Institute for, lxxxix

Babies, Books on, cxxxv
Battle, Cottage Nurses for, v
Bedford Institute, Annual Meeting of, cxix
Belfast District Nursing Society, cvii
——— Nurses, xxix
——— Nurses' Home, xlvii, lxxxiii
Berrywood Asylum, Amusements at, lxxvii
——— Attendants at, lxii
Biblewomen and Nurses, lxxvii
Birmingham City Asylum, A Nurse's Cruelty at, lxxvii
——— District Nurses, cvii
Bognor, House of Rest at, i
Bolton Fever Hospital, Scandal at, ci
Booth, "General," on Hospitals, xciv, clxv
Braintree Board of Guardians and Inadequate Nursing, lix
Brassey Holiday Home, xxi
——— at St. Leonard's, ci
Breach of Promise and Nurses, cxxxi
Brentford Guardians and Appointment of a Night Nurse, lxxi
Bridport Nurse Society, xxix
Brimsby Ghost, The, lxxvi, lxxxii
Brisbane, Nurse Training School at, lxxxiii
Buenos Ayres, Nursing at, xxviii
Burdett, Mr. H. C., on Nurses, xxix
——— Presentation to, by the First Thousand, cxlvi
Burton Nursing Institution, Report of, cxxxi
Butterworth Medal, xci
Buxton, A District Nurse for, lix

California, Nursing in, i
Canterbury Institute, Annual Report of, cxiii
Catholic Nurses, Roman, xxxv
Charing Cross Hospital, Medal for Nurses of, lxi
Charities, Love Betwixt, cxxxvii
Cheltenham, District Nurses at, cxxxi
Children, A Voice from the, xxxiv
Christmas, Keeping, lxxx, lxxxvii, xcii, xcv
——— Legend, A, lxix
——— Parcel, Distribution, lxxiv
Constantinople, Lepers at, liii
Cornwall, District Nursing in, xli
Cottage Nurses, ix, xxxii
Coventry Nursing Association, ci
Cumberland Infirmary, Nursing Institute to be started at, cxxxi

Denbigh, A District Nurse to be Started, cxix
Diaphragm, Ode to the World Below the, c
Dinner Hour, The, Suggestions as to the Time of, cxvii
Diphtheria and the Use of Brandy, lxxv
District Nurses' Case Book, ix, xxxi
Domestic Medicine in the Eighteenth Century, lii, lviii
Donegal, Industrial Development in the Wilds of, lxxxix
Dundee Nurses, xxix

Eastern Fever Hospital Scandal, cxxxi, cxxxiii, cxxxix, cxliv
East London Nursing Society, cxxxvii
Edinburgh Royal Infirmary Nurses' Home, ix
Egypt, English Nurses in, xxiii
Electricity, Medical Applications of, xcii, xcviii
——— The Institute of Medical, xliv
Energy, cxv
Eva, Sister, on Scenes in the Life of a Nurse, cxvii

Fever Hospitals, lix, lxxv, xciii, cxxxi
——— Provincial, lvii
Fitzgerald, C. E., M.D., on Physiology and Hygiene for Home Nursing, cxvi
Floors, Slippery, in Hospitals, The Dangers of, cxxiii
Frome Nurses' Home, ci

Gibraltar, New, cxli
Glasgow Royal Infirmary, lxxxiii
——— Sick Poor and Private Nursing Institution, xliii
Guardians at Hackney and Sixteen-year old "Nurses," cxxxvii
Guy's Hospital Before the Lords' Committee, civ

Haggerston Nursing Societies, cxxxvii
Hall, New District Nurses' Home at, liii
Hampshire Nurses' Institute, Annual Meeting, cxix
Hare-lipped Baby, The, xxii
Harper's Register of Nurses, i
Hart, Mrs. Ernest, on Work in Donegal, lxxxix
Hilary, Nurse, cvi, cxii, cxviii, cxxiv, cxxx
Hints for Nurses, Practical, xiv
Holiday, A Winter's, cx, cxxviii
Home Nursing, Mrs. Franks on, cxxxiii
Homerton Fever Hospital, Scandals at, xlvii
——— Ratepayers on, lix
Hospital Memories, xlvi
——— Scandals, Journalists as Advertisers of, ci
Hostel for Nurses, xiii, xli

Impromptu, c
India, Nursing in, i
Infected Clothing by Parcels Post, cxliii
Infection, Nurses and, xciii
Infectious Hospitals, cxi
Insanity, Sketches of—
XIII. Alcoholic Insanity, vi
XIV. Management of the Insane, x
Inventions, Nurses', xxxv
Ipswich, Epidemic of Measles at, xcv
Irish Board of Guardians, An, and Nursing, vii
——— Guardians and Nursing, xxxv

Johannesburg, xxxii, xxxviii
Journalists as Advertisers of Hospital Scandals, ci
Jubilee Institute, Report of, cxliii

Keeping Christmas, cvii
Kent Nursing Institution Medal, xcvii
Kimberley Hospital, A Night Experience at, cxxxiv
Kingston Nursing Association, Annual Meeting, cxxxi

Ladies' Committees, lxvii, lxxxvi
Ladies on Committees, xxvi, xxix, xlv
Leeds District Nurses, Annual Meeting of, cxiii
Lectures, ix, xxvi
Leicester Infirmary, Religious Strife at, xli
Lepers at Constantinople, liii
Liverpool Jubilee Institute, Inaugural Festival of, lxxxix
——— Northern Hospital, Library for Nurses presented to, lxxxix
——— Nurses' Training School Medal, ciii
London Hospital, v, viii, xii
——— and its Nurses, Report of Sub-Committee on, liv
——— Mrs. Hunter and her Impeachment of the, liii
——— Nursing Certificate, cxxvii
——— the Medical Staff and its Matron, xxxiii
——— the St. James's Gazette on the, lix
Longton Sick Nursing Association to be Formed, cxliii
Lords' Committee, the, civ
Lunatic Asylums, Nursing in, liii

Macclesfield Nurses, v
Male Nurses, iii, xvi, xxi, xxvi, xxxii, xxxviii, xlv, lxvii
Manchester Nursing Institute, Annual Meeting of, lxv
Marsden, Miss Kate, and the Lepers, ix
Mary Adelaide Letter, lxxvii
——— Medal, lxxiii
Massage, Nurses' Guide to, lxxxviii
Middlesex Hospital Nursing Institute, cxxv
Midwife in Trouble, A, xcv
——— or Charwoman? i
Midwives' Bill, Opposition to the, cix
——— Necessity for Registration, xiii
——— Registration Bill, Ladies on the, cxxv
Miles, A., M.B., on Surgical Ward Work and Nursing, xviii, xxiv, xxx, xxxvi, xlii, xlviii, lx, lxxii, lxxviii lxxxiv, xcvi, cii, cxiv, cxx, cxxvi, cxxxviii, cxliv
Morgan, J. S., the Family of, give £10,000 to Pension Fund, lxvi
Mothers, Advice to, xi

New York Training School for Nurses, Report of, lxxi
Night, An Eventful, xxviii
Nightingale, Miss, cxxv
Norfolk and Norwich Hospital, Nurses at, xvii, xxvii
Nursery Health Tracts, xi
Nursery, the Trained Nurse in the, xliii, l, lvii, lxxv
Nurses' Agency, a Trained, v
——— and Infection, xciii, xcix
——— Club, General Meeting of the, cvi
——— Conversazione, liii
——— Co-operation, xxiv, lxxvii, xc, cvii
——— Food, xlvii
——— Hours of Work, and Relaxation, xxix
——— Hours, lxiii
——— Missionary Association, xli
——— on their Travels, A Winter Holiday, lxii lxx, xciv
——— the Diet of, lxii
Nursing, Suggested Foundation of a Faculty of, lxxxiii
——— Heroines, xlvii
——— Mirror, Enlargement of the, xvii
——— Mirror, Ode to the, lxiv
——— Reason for Popularity of, xv
——— Theory and Practice of, xxxvii
——— Work in 1890, lxxi

Older Nurses, the Care of, xvi
Oldham Corporation, Sanitary Committee, of and Sick Nurses, xcv

Paddington Green Children's Hospital, xciii xcix, cxxxv
Palmer, Miss, Organises a Bazaar at Ashton, lxxxiii
Paris, Private Nursing in, xx
Patients, the Lifting and Moving of, ii
Paupers and Beating, cxxxvii
Pension Fund, A New Year's Gift to, lxxiv
——— A Nurse on the, xciii
——— Fourth Annual Report of, cxxxii
——— Gift of £10,000 by Family of J. S. Morgan, lxvi
——— Mary Adelaide, Letter on the, v
——— Mr. Burns on the, cxl
——— Presentation, ci
——— by the First Thousand, cxlvi
——— Some Particulars as to, cv
——— The General Bonus Fund of the, li
——— for Servants, A, cxxix
Perth Nursing Society, xcv
Phillips, Mrs., Presentation to, lxxiv
Prayer-Books for Patients, xxxviii
Prince, Princess, and People, xxxvii
Probationer, The Special, xxi, xxvi, xxxv, xxxviii
Probationers and Attacks on Hospitals, lxxv
——— Suggested to be Taught in Greater Numbers, lxxxiii

Queen Victoria Nurses, Annual Meeting of Scottish Branch, lxv

Queen's Badges, Presentation of the, cxxv

——— Nurses, The Roll of, lx[illegible]

Quetta Home, i

Ranyard, Mrs., Nur es of, lxxvii
Ray, Sister Ruth, A Heroine, xv
Reading for the Sick :—
To Hospital Patients, xxxvii
The Duty of Cheerfulness, xlix
Preparing for Christmas, lxi
Snow, lxxiii
Christ in our Hearts, lxix
The Old Year, lxxix
Encouragement, lxxxvi
Gratitude, xci
The Sun, xcvii
To the Young, ciii
Forsaken, cxxvii
Help in Suffering, cxxxix
Bad Nights, cix
The Invitation, cxlv
Rentoul, Dr. R., on the Dignity of Woman's Health, cxvi
Reviews :—
New Life: Its Genesis and Culture (O'Neil and Barnett), xi
Nursery Health Tracts, xi
Handbook for the Instruction of Attendants on the Insane, xxxvii
Prince, Princess, and People (Burdett), xxxvii
Theory and Practice of Nursing, The (Dr. Lewis), xxxvii
The Nurses' Guide to Massage (S. Hyde) lxxxviii
The Care of the Sick (Dr. T. Billroth) lxxxviii
Physiology and Hygiene for Home Nursing, cxvi
Nursing and Hygiene, cxvi
The Dignity of Woman's Health, cxvi
Scenes in the Life of a Nurse, cxvii
Babyhood, cxxxv
Our Baby (Mrs. Langton Hewer), cxxxv
Confidential Chats with Mothers (Mrs. Bowditch), cxxxv
Roberts', Lady, Nurses in India, civ
——— R. Lawton, M.D., on Nursing and Hygiene, cxvi
Rose Gertrude, Sister, The Fall of, xvii
Royal Red Cross, The, xlix
Rugby District Nursing Association, xxiii
Rumbles, cxxxvi
Rural Nursing Association, lix, xcv

St. Bartholomew's Hospital before the Lords' Committee, civ
St. Helena Home, End of Squabbling at, lxxvii
——— Facts Respecting, lxxi
St. John the Divine, the Sisters of, Annual Report, cix
St. Thomas's Hospital before the Lords' Committee, civ
Servants of the Poor, Homerton, xiii
Sewing Competition, xcvii
Sick Nurse, The, xxxv
—— Nurses, Home for, xxiii
—— The Care of, lxxxviii
Six Months in a Hospital, iii, x, xiv, xix
Soldiers' Wives as Nurses, i
Southend, A New Institute for Invalids at, cxiii
Suffolk General Hospital, Management Attacked by a Six Months' Probationer, lxv
Sunderland Nursing Institute, cvii
Surgical Ward Work and Nursing, Lectures on :
I. Antiseptics, xviii
II. Preventing Sepsis, xxiv
III. and IV. Antiseptics in Common Use, xxx, xxxvi
V. Wool and Lint, xlii
VI. Dressings, xlviii
VII. Preparing for an Ordinary Dressing, lx
VIII. Wounds and Cleanliness, lxxii
IX. The Dressing of Wounds, lxxviii
X. The Spray Dressing, lxxxiv
XI. ——— xcvi
XII. Management of Surgical Operations, cii
XIII. Operations, cviii
Surgical Ward Work and Nursing, Lectures on :
XIV. Ligatures and Tourniquets, cxiv
XV. Dressing Trays and Anæsthetics, cxx
XVI. Dangers of Chloroform and their Treatment, cxxvi
XVII. Preparation of Patient for Operation, cxxxviii
XVIII. After Operation, cxliv

Taunton Victoria Institute, xxix
Tetanus, xliv
Theatricals, xxxiv
Thirty Pounds a Year, On, lxiv
Through the Hospital, cxlviii
Truro, Nurses' Home, cvii, cxli
——— A Correction, cxxiii
Tynemouth Infirmary, xvii, xxvi
Typhoid, The Death Rate in, cxi, cxxiii

Understandings, Our, xl, xlvi

Vancouver Christmas at, lxviii

Walmsley, Dr. F. H., on Insanity, vi, x
Wardmaids in London Hospital, Notes on, lix
——— instituted at Middlesex Hospital, liii
Watford District Nurses, xiii
Winchester, District Work at, cxxxi
Winter's Holiday, A :—
IV. In Spain, cxxi
Woman's Life, A, cxix
Worcester City Institution, cxxv
Words of Consolation :—
For the Aged, xxv
The Great Physician, xix
Love, xxxi
Workhouse Infirmaries, Beating of Patients at, ci
——— Northern, xiii
——— Infirmary Nursing Association, Annual Report of, cxiii
Workhouses, Christmas Fare in, lxxxvi

### NOTICE TO CORRESPONDENTS.

All MS., Letters, Books for Review, and other matters intended for the Editor should be addressed THE EDITOR, THE LODGE, PORCHESTER SQUARE, LONDON, W.

The Editor cannot undertake to return rejected M.S., even when accompanied by stamped directed envelope.

### Notice to Advertisers and Subscribers.

All Advertisements, Orders for Copies of THE HOSPITAL, and Business Communications should be addressed to The Publisher, *not to the Editor*, THE HOSPITAL, 140, Strand, W.C.

### Bound Volumes of "The Hospital."

Vol. VI., containing full page portraits of T.R.H. the Prince and Princess of Wales, and Miss Florence Nightingale, is obtainable at 4s., post free.

Orders will be received for Vol. VII., 4s. post free. Cases for binding may be had of the Publisher, at 1s. 6d. each.

### To Residents, Secretaries, and Matrons.

The Editor will be glad to have the Regulations and each Report as issued by Asylums, Hospitals, Convalescent and Nursing Institutions, as well as those of other Charities, and an early copy in duplicate of all Appeals and Festival Notices, etc., addressed to *The Editor, The Lodge, Porchester Square, London, W.* Any superintendent, secretary, matron, or other chief official who regularly sends such information may be registered, on application to the Publisher, to receive free of cost a cover for binding THE HOSPITAL in half-yearly volumes.

### BURDETT'S HOSPITAL ANNUAL FOR 1890

**Is now ready, and may be obtained of the Publisher, THE HOSPITAL Offices, 140, Strand, W.C., price 2s. 6d. limp boards, or 3s. 6d. cloth. All orders must be accompanied by a remittance.**

### Death Certificates and Murde .

### Students' Column.

### UNIVERSITY OF DURHAM.

FACULTY OF MEDICINE.

At the Convocation holden on Tuesday, September 30th, 1890, the following were recommended for the Degree of Doctor in Medicine for practitioners of fifteen years' standing, viz. :—

Chapman, C. W., M.R.C.P. Lond., M.R.C.S. Eng.; Gibson, W. J., L.R.C.S. Ire., L.K.Q.C.P. Ire., L.M.; Harvey, J. S. S., M.R.C.S. Eng., B.Sc., Licentiate of Medicine, Paris; Martin, R. J., F.R.C.S., L.R.C.P. Ed., M.R.C.S. Eng.; Milroy, A., L.R.C.P. Ed., L.F.P.S., D.P.H., Glasgow; Plowright, C. B., M.R.C.S. Eng., L.R.C.P. Ed.

And the following have been recommended for the degree of M.D., viz. :—

Arnison, W. D., M.B., B.S. Durh., M.R.C.S. Eng., L.R.C.P. Lond.; Blacker, A. B., M.B., B.S. Durh., M.R.C.S., L.S.A., L.R.C.P. Lond.; Bowmaker, E., M.B., B.S. Durh.; Burton, F. H. M., M.B., M.S. Durh., M.R.C S. Eng.; Davis, I., M.B., B.S. Durh., D.P.H.; Dixson, C. F. L., M.B. Durh., M.R.C.S. Eng.; James, J. T., M.B. Durh., F.R.C.S. Eng., M.R.C.S., L.R.C.P., L.S.A.; Sweetapple, H. A., M.B., B.S. Durh.; Wilson, J. H., M.B., B.S. Durh.

And the following have been recommended for the degree of Bachelor of Medicine (M.B.), viz. :—

Honours—*First Class*: Beattie, T., College of Medicine, Newcastle-upon-Tyne.—*Second Class*: Morland, C. H. D., M.R.C.S., L.R.C.P., St. George's Hospital; Martin, A. M., College of Medicine, Newcastle-upon-Tyne.

Pass List.—Adkins, P. R., St. Thomas's Hospital; Averill, C., M.R.C.S., L.S.A., St. Bartholomew's Hospital; Alderson, F. H., L.R.C.P. Lond., Middlesex Hospital; Brown, R. C., College of Medicine, Newcastle-upon-Tyne; Buckham, T., College of Medicine, Newcastle-upon-Tyne; Cross, G. F., College of Medicine, Newcastle-upon-Tyne; Eardley-Wilmot, C. C., St. Bartholomew's Hospital; Harris, F. S., M.R.C.S., L.R.C.P., London Hospital; Hewer, A. A., St. Bartholomew's Hospital; Melville-Davidson, W., College of Medicine, Newcastle-upon-Tyne; Plummer, S. W., College of Medicine, Newcastle-upon-Tyne; Stanley, C. J., M.R.C.S., L.R.C.P., King's College, London; Stokoe, B. T., College of Medicine, Newcastle-upon-Tyne; Turnbull, W. H., College of Medicine, Newcastle-upon-Tyne.

The following have been recommended for the Degree of Bachelor in Surgery (B.S.), viz. :—

Averill, C., M.R.C.S., L.S.A., St. Bartholomew's Hospital; Beattie, T., College of Medicine, Newcastle-upon-Tyne; Brown, R. C., College of Medicine, Newcastle-upon-Tyne; Buckham, T., College of Medicine, Newcastle-upon-Tyne; Cross, G. F., College of Medicine, Newcastle-upon-Tyne; Eardley-Wilmot, C. C., St. Bartholomew's Hospital; Hewer, A. A., St. Bartholomew's Hospital; Martin, A. M., College of Medicine, Newcastle-upon-Tyne; Melville-Davidson, W., College of Medicine, Newcastle-upon-Tyne; Morland, C. H. D., M.R.C.S., L.R.C.P., St. George's Hospital; Plummer, S. W., College of Medicine, Newcastle-upon-Tyne; Stokoe, B. T., College of Medicine, Newcastle-upon-Tyne; Turnbull, W. H., College of Medicine, Newcastle-upon-Tyne.

### General Charities.

[This Column is devoted to an account of work of charitable institutions other than Medical Charities. The Editor will be glad to receive reports or news of work at 140, Strand. *Philanthropy* should be plainly written on the envelope.]

**THE PHILANTHROPIC SOCIETY'S FARM SCHOOL AT REDHILL** states in its report that out of 124 boys disposed of at home 102 are doing well, two are doubtful, nine have been reconvicted, and the result of three cases is unknown; of 117 emigrated 108 are doing well, and only five reconvicted. These lads have all been convicted of crime, and such a report is admirable testimony to the efficiency of the management. So much, too, for those who hold that penal discipline is the only effectual means of reforming the youthful offender, the Farm School at Redhill shows what healthy employment and new ideas can do for a so-called criminal.

Mr. J. W. C. Fegan, of the **BOYS' HOME, SOUTHWARK,** who does so much good amongst street arabs, sends us a number of little books relating to his work, and a special appeal for help, but no report or balance-sheet. Balance-sheets in these days are complex things to arrange and set in order, no doubt; but they are an absolute necessity as a guarantee of good faith, and if you can come to determine that for 5s. a dinner can be given to thirty waifs, or for £2 a waif can be completely outfitted, or for £15 a waif can be sent all ready to Canada, it scarcely seems a much more difficult matter to determine the outlay of the whole affair. We expect a hospital to render a full and particular account of its use of public funds; we expect no less of charitable institutions.

**THE VALENTIA ISLAND FISHERMEN.**—An appeal reaches us from as far away as the little island of Valentia, off the coast of Kerry; no very rich spot, as everybody knows, and the appeal is worthy of help. Every week in the spring this island is frequented by about four hundred fishermen of Irish, Scotch, and Isle of Man fishing fleets. The men put in on Saturday morning and stay till Monday afternoon, and literally the only place of shelter they can find is the public-house. Up to last year an old office was used as a reading-room, to which as many men went as could get in; but this last spring the office was wanted for other purposes, and the loss of it was severely felt, and the men who did not sit in the public-house sat about in ditches, no matter what the weather. What is wanted is a cheap substantial building containing a reading-room and recreation-hall, which would cost about £250 to build, and for which the Knight of Kerry has given a site, and it is earnestly hoped that it may be able to be built ready for next spring. We should think a night shelter ought to be included in this building. Sailors have plenty of privations and hardships, and life on a fishing boat is not unmixed delight, and so we do earnestly ask any of our readers who can to help these men to get a covering before spring comes. We will gladly forward any subscriptions however small, or any contributions may be sent to Anna J. Delap, The Parsonage, Valentia Island, Co. Kerry.

### Scraps and Gleanings.

Alderman Smith has presented an ambulance carriage to Scarborough.

The Duchess of Montrose will open the Glasgow Cancer Hospital on October 13th.

The Sanitary Institute has arranged a series of lectures to commence on October 3rd.

Three men were killed in New York last week by accidental contact with electric wires.

Lady Clifton lately presented ambulance certificates to 8 men and 14 women at Blackpool.

Dr. Alfred Crespi writes an article in *The Gentlemen's Magazine* on "Cocoa and Chocolate."

Sir Morell Mackenzie writes an article in the *Contemporary Review* on "The Use and Abuse of Hospitals."

Scarlatina being prevalent in Liverpool, two hospital tents have been erected at Dingle Point for the extra patients.

Typhoid fever has broken out amongst the French troops at St. Nicolas; it is said to have arisen through bad meat.

Hartlepool lately held a very successful demonstration by friendly societies in aid of the hospital. Over £46 was collected.

Bideford can congratulate itself on the enormous success of its bazaar in aid of the infirmary; over £1,000 was raised.

The Governors of St. Peter's Hospital for Stone have received a draft for £1,000 for the afflicted poor from Mr. W. H. Johnson.

The Queen of Roumania has taken great interest in our English charities: she attended a fête in aid of the Llandudno Cottage Hospital.

Books for the blind are very dear; how is it then that more young ladies do not learn to emboss these books for the use of the poor blind?

The governors of the Birmingham General Hospital, on September 26th, approved a scheme for the entire removal of the hospital to another site. This is important news.

Dr. Conry and Dr. Walker, of Salford Hope Hospital have both been called upon to resign. Our readers will remember the circumstances of the last of the many Hope Hospital scandals. Let us hope it is the last.

